# Neurosurgical Care during the COVID-19 Pandemic in Central Germany: A Retrospective Single Center Study of the Second Wave

**DOI:** 10.3390/ijerph182212034

**Published:** 2021-11-16

**Authors:** Caroline Sander, Nikolaus von Dercks, Michael Karl Fehrenbach, Tim Wende, Sebastian Stehr, Dirk Winkler, Jürgen Meixensberger, Felix Arlt

**Affiliations:** 1 Department of Neurosurgery, University Hospital Leipzig, 04103 Leipzig, Germany; caroline.sander@medizin.uni-leipzig.de (C.S.); michael.fehrenbach@medizin.uni-leipzig.de (M.K.F.); Tim.Wende@medizin.uni-leipzig.de (T.W.); Dirk.Winkler@medizin.uni-leipzig.de (D.W.); juergen.meixensberger@medizin.uni-leipzig.de (J.M.); 2 Department for Medical Controlling, University Hospital Leipzig, 04103 Leipzig, Germany; Nikolaus.vonDercks@medizin.uni-leipzig.de; 3 Department of Anesthesiology and Intensive Therapy, University Hospital Leipzig, 04103 Leipzig, Germany; sebastian.stehr@medizin.uni-leipzig.de

**Keywords:** COVID-19, corona virus, neurosurgical care, neurosurgery, unplanned readmission, index diagnosis, surgical procedure

## Abstract

The healthcare system has been placed under an enormous burden by the SARS-CoV-2 (COVID-19) pandemic. In addition to the challenge of providing sufficient care for COVID-19 patients, there is also a need to ensure adequate care for non-COVID-19 patients. We investigated neurosurgical care in a university hospital during the pandemic. We examined the second wave of the pandemic from 1 October 2020 to 15 March 2021 in this retrospective single-center study and compared it to a pre-pandemic period from 1 October 2019 to 15 March 2020. Any neurosurgical intervention, along with patient- and treatment-dependent factors, were recorded. We also examined perioperative complications and unplanned readmissions. A statistical comparison of the study groups was performed. We treated 535 patients with a total of 602 neurosurgical surgeries during the pandemic. This compares to 602 patients with 717 surgeries during the pre-pandemic period. There were 67 fewer patients (reduction to 88.87%) admitted and 115 fewer surgeries (reduction to 83.96%) performed, which were essentially highly elective procedures, such as cervical spinal stenosis, intracranial neurinomas, and peripheral nerve lesions. Regarding complication rates and unplanned readmissions, there was no significant difference between the COVID-19 pandemic and the non-pandemic patient group. Operative capacities were slightly reduced to 88% due to the pandemic. Nevertheless, comprehensive emergency and elective care was guaranteed in our university hospital. This speaks for the sufficient resources and high-quality processes that existed even before the pandemic.

## 1. Introduction

The coronavirus pandemic has had a huge impact on healthcare systems worldwide. The healthcare system has not only been burdened by the increase in the number of infections with the coronavirus-19 (COVID-19) but also had to continue to provide emergency and urgent care for non-COVID-19 patients [1,2]. Thus, the healthcare system has faced an enormous challenge. This particularly affects neurosurgery, as many diseases in this field have an emergency character. Neurosurgical diseases are characterized by enormous heterogeneity, complexity, rapid tendency to undergo neurological deterioration and urgent care character [3]. A large number of neurosurgical operations have still been required during the pandemic [4].

There has been an increased demand for ventilators and intensive care resources during the pandemic. These resources were taken away from non-COVID-19 patients. Thus, triaging and prioritization of patients in the non-COVID-19 area and deferral of elective procedures was required [5].

An uncertainty resulted, therefore, regarding the impending undertreatment of non-COVID-19 cases in favor of the redistribution of COVID-19 patients under focus [6]. Consequently, universal recommendations have been necessary. Numerous guidelines and recommendations for prioritizing, shifting, and structurally utilizing tightening resources during the pandemic were published [7,8,9].

Accordingly, we looked at the period of the second wave of the pandemic in Saxony, Germany, compared to the pre-pandemic time regarding surgical procedures, complications, and readmissions in our neurosurgical department at a university hospital. 

Unplanned readmissions consume potentially preventable resources, incur costs, and compromise patient satisfaction. Therefore, investigating the causes and predictors of unplanned readmissions is essential to achieve quality improvement. Unplanned readmissions represent a marker of quality, [10,11,12] and also allow the estimation of patient care during the COVID-19 pandemic. To the best of our knowledge, we are the first to investigate the impact of the COVID-19 pandemic on the quality of neurosurgical care measured by surgical procedures, complications, and unplanned readmissions.

## 2. Materials and Methods

The internal review board of the Medical Faculty of the University Hospital Leipzig agreed to the retrospective data analysis (167/18-ek). All neurosurgical patients who underwent neurosurgical surgery during the period of the second wave of the COVID-19 pandemic in Saxony, Germany, from 1 October 2020 to 15 March 2021 (pandemic group) were included in this retrospective monocentric study. This patient group was compared to a comparison group of neurosurgical patients who had surgery from the period 1 October 2019 to 15 March 2020 (pre-pandemic group). Patient informed consent was not required according to the approval of the ethics committee. Patient criteria, hospital- and surgery-related factors, perioperative complications, and unplanned readmissions 30 days after discharge were analyzed. If multiple surgeries were performed, the index surgery, the main surgery in that inpatient stay, was listed. The first set of ‘index admission’ diagnoses contained all neurosurgical diseases according to the ICD-10 GM list. The patients were grouped into ‘neoplasm,’ ‘hydrocephalus,’ ‘traumatic head injury,’ ‘vascular,’ ‘functional disorder,’ ‘degenerative spine disease,’ and ‘others’ (e.g., abscess, trigeminus neuralgia). The patient clinical complexity level (PCCL) was defined via the effective assessment ratio of the German diagnosis-related groups’ coding level, which integrates the technical procedures and the patient’s secondary diagnoses. Causes for readmission were noted as: (1) surgical complications, (2) medical complications, (3) diagnosis-related complications (e.g., progression of tumor, hydrocephalus), (4) neurological decompensation (e.g., stroke, seizure, neurologic symptoms), (5) pain management, and (6) others (e.g., unrelated diagnoses, admissions due to missing home care). Categories of readmission were defined as: (1) preventable reasons (e.g., surgical site infection (SSI), cerebrospinal fluid (CSF) leak, postoperative hemorrhage, nosocomial infection, postoperative pain, falls), (2) reasons related to the natural progression of the disease (e.g., occlusive hydrocephalus, seizures), (3) reasons despite best practice (e.g., stroke), and (4) unrelated reasons according to the study by Shah et al. [3].

Statistical analysis was performed with IBM SPSS Statistics 25.0 software (IBM, Armonk, New York, NY, USA). The associations between continuous variables were examined using the *t*-test for normal distribution and the Mann–Whitney U test for variables without normal distribution. Categorical variables were compared by employing the Fisher exact test. Continuous variables were described using mean and median values, while categorical variables were described with counts and frequencies. A two-tailed *p* value < 0.05 was considered to be statistically significant. 

## 3. Results

### 3.1. Surgical Procedures

**Figure 1:**
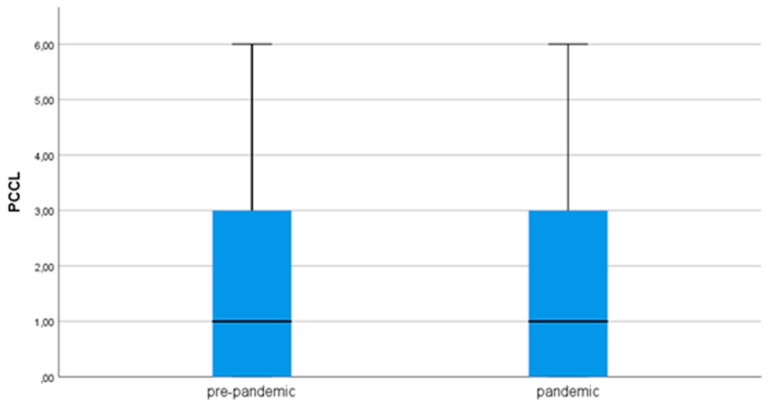
Median PCCL pandemic vs. pre-pandemic patient group.

A total of 535 patients were included in the COVID-19 pandemic group, and a total of 602 operations were performed. By contrast, 602 patients were treated in the non-pandemic group, and a total of 717 operations were performed. Thus, there was a decrease of 67 patients (reduction to 88.87%), and a total of 115 fewer surgeries (reduction to 83.96%) were conducted in the COVID-19 pandemic group. In addition, the operating room capacity had been reduced to 88% due to higher intensive care unit resources necessary to take care of critically ill COVID-19 patients during the pandemic. Analysis stated that the two groups, pandemic and pre-pandemic, did not differ in the patient characteristics (see Table 1). The patient groups were similar in terms of age, gender, and index diagnosis groups. When we analyzed the admission modalities, we observed identical frequencies of elective and emergency admissions in the pandemic and the pre-pandemic period. Discharge modalities were also identical in both groups. When the operation-dependent factors were examined, a homogeneity was found between the two study groups. The number of operations, the length of surgery, and the timing were identical. The PCCL was identical during the pandemic and pre-pandemic, and the duration of ventilation did not change during the pandemic compared to the pre-pandemic (Figure 1). With regard to the index diagnosis groups, a categorization of the neurosurgical diseases according to the ICD-10 GM list was performed. Fewer intracranial neurinomas, cervical spinal stenoses, and peripheral nerve injuries were treated surgically during the pandemic. By contrast, no diagnostic group was represented more frequently in the coronavirus group (see Table 2).

### 3.2. Complications

**Figure 2:**
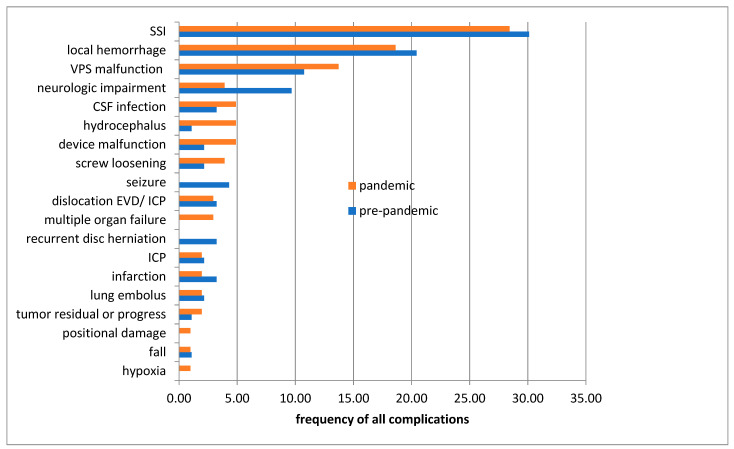
Perioperative complications in the pandemic and pre-pandemic patient group. ICP, intracranial pressure; EVD, external ventricle drainage, CSF, cerebrospinal fluid; VPS, ventriculoperitoneal shunt; SSI, surgical site infections.

In the next step, we looked at the perioperative complications. Figure 2 presents the different complications. There is a homogeneity of the two groups with no significant difference. The most frequent complications registered in both groups are SSIs followed by local postoperative bleeding.

### 3.3. Readmissions

**Figure 3:**
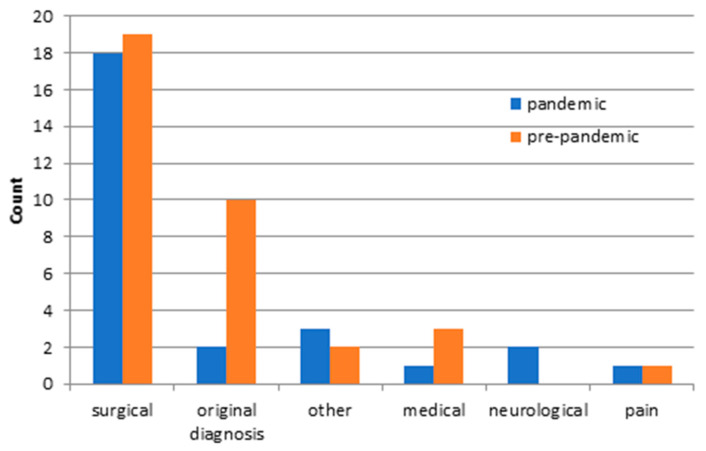
Causes for unplanned readmission in the pandemic and pre-pandemic patient group. Other: unrelated diagnoses, admissions due to missing home care.

**Figure 4:**
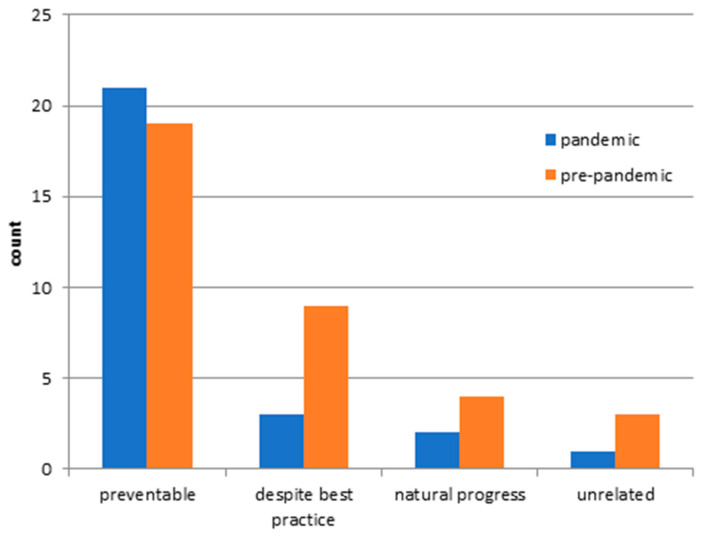
Readmission category in the pandemic and pre-pandemic patient group.

Finally, we examined the unplanned readmissions in both groups. The unplanned readmission rate was 5.05% (27 cases) during the pandemic, while the pre-pandemic readmission rate was 5.81% (35 cases). The detailed reasons for readmissions were without statistical difference for both groups (see Supplementary Table S1). A closer look at the detailed reasons for readmission indicated that SSIs were responsible for most of the unplanned readmissions. There is no significant difference (*p*-value = 0.390), but there is a trend towards a reduction in the pandemic group (10 cases pre-pandemic vs. 5 cases pandemic). A significant difference between the reasons for readmission was not found. Further factors regarding the unplanned readmission group with patient-related and treatment-dependent factors are shown in Table 3. There were no significant differences for either study group. Furthermore, there were similar frequencies for the pandemic and the pre-pandemic group regarding the causes for readmissions (Figure 3). The surgical causes predominated in both groups. A progress of the underlying disease was responsible for the majority of pre-pandemic unplanned readmissions, whereas new medical complications caused the majority of unplanned readmissions in the pandemic group. In the following, the readmissions were divided into categories (Figure 4). There was no significant difference in readmission categories for either study group. Most unplanned readmissions were for the pandemic and pre-pandemic study group classified as ‘preventable,’ followed by ‘despite best practice’. 

## 4. Discussion

Our study demonstrated the differences and similarities of patients treated neurosurgically during the COVID-19 pandemic compared to pre-pandemic in a retrospective, monocentric study. 

### 4.1. Surgical Procedures

Many similarities were found between the study groups in terms of patient characteristics and treatment-related factors. For instances, we revealed a statistically similar distribution in terms of age, sex, and index diagnosis groups of patients during the COVID-19 pandemic compared with the pre-pandemic period. However, there were also significant differences between the study groups. Significantly fewer intracranial neurinomas, peripheral nerve lesions, and cervical spinal stenosis were surgically treated and hospitalized during the pandemic. These three admission diagnoses are among the highly elective and postponable procedures. In a comprehensive survey of neurosurgical centers in Europe during the early part of the COVID-19 pandemic in March 2020, decreases in elective craniotomies and intermediate and regular neurosurgical beds were reported in the majority of centers. The decrease in elective procedures was necessary to ensure medical care for the increasing number of COVID-19 patients. Resources in the hospital such as intensive care beds and medical staff are limited. Therefore, the need for prioritization and triage of procedures and emergencies has emerged to adequately care for both COVID-19 patients and neurosurgical cases [4]. For example, a recent study found a 40% decrease in neurosurgical operations, clinical visits, and inpatient consultations, and a significant decrease in spine and endovascular procedures during the pandemic compared with the pre-pandemic [13]. Interestingly, there were also few European centers that reported no decreases in the quantity and quality of medical care of neurosurgical procedures, elective as well as emergency cases due to the pandemic [4]. Another study of neurosurgical patients during the COVID-19 pandemic in Germany found a slight decrease in patients and (1278 cases pandemic versus 1379 pre-pandemic) and surgical procedures [14].

Triaging of highly elective procedures is possible for patients who are not at risk of neurologic deficit due to the waiting time of postponed surgeries. In some cases, a stabilization and decrease of the initial symptoms during the waiting period was described for this patient collective. This brings the conservative therapy of these diseases back into the foreground [15,16]. In addition, the avoidance of patients with a low symptom burden before hospitalization or the initial favoring of conservative therapy could also explain the decrease in elective admissions causally. Differential prioritization and rationing in neurosurgical centers across Europe were demonstrated [4]. Some centers were able to provide emergency care, depending on the region, whereas others also described an uninterrupted elective program. Here, the enormous difference in the volume of neurosurgical procedures performed was attributed to the varying severity of the COVID-19 burden and the resources available. Some centers had less bed capacity and a longer waiting list even in pre-pandemic times, which was exacerbated by the COVID-19 pandemic [4]. The most pandemic constraints were reported in neurosurgical centers, where regions were particularly severely affected by the COVID-19 pandemic, had large catchment areas and low resources. In addition, the number of beds and longer waiting lists for neurosurgical procedures were already present in these centers before the pandemic. As a result, there was little flexibility in adjusting COVID-19 measures related to triage, rationing, and prioritization, leading to a high degree of change in indications and services [4].

### 4.2. Emergency Admission

Some neurosurgical departments responded quickly and effectively to the redistribution of resources during the pandemic. In addition to reducing the surgical capacity, a change in indications, namely task shifting, is also an efficient way to adapt resources during the pandemic [4]. There is a division of responsibilities among the centers, with different responsibilities for emergency surgeries, elective surgeries, and, of course, the care of COVID-19 patients regarding task shifting [17]. Consequently, the catchment area of the neurosurgical departments changes during the pandemic and, therefore, the potential number of patients to treat. Interestingly, we could not register any changes in emergency cases, mortality, or night-shift surgery during the pandemic compared to the pre-pandemic time course. Despite this, a decrease in emergency neurosurgical cases, such as traumatic brain injuries, spine conditions, or chronic subdural hematomas, was noticed [18]. However, no decrease in neurosurgical emergencies or night procedures was evident in our study. We cared for statistically equal numbers of emergencies, elective procedures, and night shift procedures before and during the pandemic. These variations in the different neurosurgical centers can be explained by differences in coronavirus burden within regions, differences in resources and bed distribution, and, of course, catchment area [4]. Thus, an absolute reduction in neurosurgical emergencies per population can still be masked by an expanded catchment area, as was present in our neurosurgical center. 

### 4.3. PCCL

Considering the severity of cases during the pandemic and pre-pandemic period, there was no difference. By contrast, an increase in PCCL during the pandemic was documented in the visceral surgery patient population at the University Hospital Leipzig, which was explained by the predominance of severely ill patients in the case mix [19]. An increase in PCCL during the pandemic is attributable to the fact that primarily sicker patients with emergency indications were hospitalized. Treatments for healthier patients with elective indications were postponed or canceled according to prioritization and triage. Interestingly, no change in PCCL was evident in our study, which would have been expected from a shift in index diagnosis groups and triage. There are several possible explanations for this. First, we found no statistically significant change in the number of emergencies versus elective procedures for neurosurgical patients. Only for three index diagnosis groups, intracranial neurinomas, peripheral nerve lesions, and cervical spinal stenosis, a significant reduction of admissions during the pandemic compared to the pre-pandemic was evident. Moreover, it was striking that the neurosurgical patients already had a relatively high median PCCL. Therefore, the comparison of the different patient groups is per se difficult.

### 4.4. Complications

The type of complications and the complication rate of surgically treated patients did not change during the pandemic. The most common perioperative complications in both groups were SSIs and local hemorrhages. It should be emphasized, however, that a trend for less SSI among patients receiving neurosurgical care was evident during the pandemic, but this difference was not statistically significant. A recently published study in a large neurosurgical center in Germany described a drastic decrease in SSI after neurosurgical procedures due to the strict hygiene measures and limited visitors in the hospital [14]. It should be underlined that in our study 29 cases (28.34% of all complications in the pandemic group) versus 28 cases (30.11% of all complications in the pre-pandemic group, *p*-value 0.588) were identified. Therefore, a reliable conclusion or even confirmation of the above-cited study is not possible based on our data with the small number of cases.

### 4.5. Readmission

Finally, we examined the unplanned readmissions in the two study groups. No significant differences were found regarding the index diagnosis, the LOS, the occurrence of complications, or the PCCL of the index admission. Reasons, causes, and categories for readmission are also equally prevalent with predominantly surgical causes in both groups. We showed previously that readmission rates in neurosurgery ranged from 5.7 to 9.2%, depending on the index diagnosis [19,20]. We confirmed a similar readmission rate in this study population during the pandemic and pre-pandemic period. Furthermore, SSIs again emerged as the leading cause of readmissions. In the literature, SSIs were found repeatedly to be the most common reason for unplanned readmission [1,6,18]. The majority of unplanned readmissions in neurosurgery belong to the group of preventable readmission categories. This fact has already been described by Sander et al. in cranial and spinal neurosurgical patients [16,17]. Interestingly, a lower incidence of SSIs during the COVID-19 pandemic period was striking as a preventable category of unplanned readmission. This correlation is not statistically significant and should, therefore, only be considered hypothetically. A significant decrease in SSIs during the pandemic was described in another neurosurgical collective. Intensified hygiene measures with an increased use of disinfectants in everyday life, the mandatory use of medical or FFP2 masks, and the stricter restriction of hospital visits may explain a tendency toward a decrease in the number of germs circulating [4].

## 5. Conclusions

This study investigated the quality and quantity of medical care provided to neurosurgical patients during the COVID-19 pandemic. Ours was the first study to demonstrate only minor statistical differences among neurosurgical patients during the pandemic compared with pre-pandemic. Thus, in terms of patient characteristics such as age, sex, and PCCL, we showed that the study groups did not differ. In addition, most treatment-related factors such as type of admission and type of discharge, length of stay, certain index diagnoses, and surgical procedures were statistically the same. It is important to emphasize that there were no differences in perioperative complications, predominantly SSI and local hemorrhage, for both study groups. Furthermore, the COVID-19 pandemic did not affect the rate of unplanned readmissions (5.08% vs. 5.81%, pandemic vs. pre-pandemic), which is known to be a quality measure of medical care in health care services. Causes and reasons for readmission were statistically similar for both study groups. There are only minor statistical differences between the neurosurgical patients in the COVID-19 pandemic and pre-pandemic period. Some highly elective, postponable procedures, such as intracranial neurinomas, peripheral nerve lesions, and cervical spinal stenosis, were treated with fewer hospitalizations during the COVID-19 pandemic. The decrease in highly elective procedures can be explained by prioritization, rationing, and triage in favor of critically ill patients. Moreover, during the COVID-19 pandemic, a decrease in SSI as perioperative complications and as unplanned readmission cause in the corona was recorded. However, it should be emphasized that it was only apparent as a trend and did not become statistically significant.

The absence of massive limitations due to the COVID-19 pandemic in our neurosurgical center can be assumed due to a good resource distribution and structuring already existing before the pandemic with a simultaneous expansion of the catchment area.

In summary, we demonstrated that broad cross-diagnosis care of neurosurgery patients was provided at our hospital, even during the COVID-19 pandemic.

## Figures and Tables

**Table 1:** Patient demographics and operation procedures for the pandemic and pre-pandemic patient group.

Total Group N = 1137	Pandemic		Pre-Pandemic		
	Median; Range	N (%)	Median; Range	N (%)	*p*-Value
		535 (47.05)		602 (52.95)	
Age, yrs	56.77(60; 0–95)		57.23 (60; 0–93)		0.749
Gender, female		255 (47.66)		279 (46.35)	0.350
LOS, d	12.24 (7; 0–87)		12.98 (7; 0–83)		0.581
**Index diagnosis group**					
Neoplasm		159 (29.72)		183 (30.40)	0.846
Degenerative spine		135 (25.23)		152 (25.25)	1.000
Vascular		92 (17.20)		101 (16.78)	0.874
Hydrocephalus/ malformation		56 (10.47)		52 (8.64)	0.312
Functional		44 (8.22)		51 (8.47)	0.915
Trauma		40 (7.48)		40 (6.64)	0.643
Nerve		3 (0.56)		10 (1.66)	0.098
Other		6 (1.12)		13 (2.16)	0.246
**Admission category**					
Elective		299 (59.92)		367 (60.96)	0.7569
Emergency		165 (33.07)		187 (31.06)	0.5163
External hospital		30 (6.01)		46 (7.64)	0.3396
Newborn		5 (1.00)		2 (0.33)	0.2550
**Discharge**					
Home		436 (81.49 )		490 (81.40)	1.000
External hospital		40 (7.48)		51 (8.47)	0.585
Rehabilitation		31 (5.79)		32 (5.32)	0.795
Death		27 (5.05)		26 (4.32)	0.576
Patient’s discretion		1 (0.19)		3 (0.50)	0.627
**Surgery**					
Surgery time, min	112 (97;0–477)		114 (99; 0–496)		0.837
Surgery during nighttime		61 (10.13)		61 (8.51)	0.393
PCCL	1.41 (1; 0–6)		1.5 (1; 0–6)		0.390
Ventilation time, h	41 (0; 0–1728)		40 (0; 0–1041)		0.984

N, number; yrs, years; LOS, length of stay; d, days; PCCL, patient clinical complexity level; h, hours.

**Table 2:** Index diagnoses for neurosurgical admission in the pandemic and pre-pandemic patient group.

	Pandemic	Pre-Pandemic	
Index Diagnosis	N (%)	N (%)	*p*-Value
**Neoplasm**			
Primary brain tumor, malign	43 (8.04)	43 (7.14)	0.5764
Meningioma	36 (6.73)	37 (6.15)	0.7172
Secondary brain tumor	24 (4.49)	34 (5.65)	0.4190
Pituitary gland adenoma	20 (3.74)	16 (2.66)	0.3136
Unknown neoplasm	15 (2.80)	22 (3.65)	0.5039
Primary brain tumor, benign	10 (1.87)	6 (1.00)	0.3132
CNS lymphoma	5 (0.93)	9 (1.50)	0.4325
Cranial neurinoma	2 (0.37)	12 (2.00 )	0.0145
Myeloma tumor, malign	4 (0.75)	3 (0.50)	0.7126
Myeloma tumor, benign	0	1 (0.17)	1.000
**Spine disease**			
Lumbar disc herniation	60 (11.21)	49 (8.14)	0.086
Spinal stenosis, lumbar	46 (8.60)	54 (8.97)	0.8347
Cervical disc herniation	17 (3.18)	22 (3.65)	0.745
Spinal stenosis, cervical	7 (1.31)	21 (3.49)	0.0207
Spondylodiscitis	2 (0.37)	1 (0.17)	0.6039
Spondylolisthesis	2 (0.37)	4 (0.66)	0.6899
Thoracal disc herniation	1 (0.19)	1 (0.17)	1.000
**Vascular**			
ICH	35 (6.54)	42 (6.98)	0.8137
Chronic SDH	24 (4.49)	24 (3.99)	0.768
SAH	16 (2.99)	20 (3.32)	0.8656
Stroke	10 (1.87)	9 (1.50)	0.6500
Hemangioma	4 (0.75)	5 (0.83)	1.000
Aneurysm	3 (0.56)	1 (0.17)	0.348
**Hydrocephalus/Malformation**			
Hydrocephalus occlusus	28 (5.23)	19 (3.16)	0.0999
NPH	9 (1.68)	10 (1.66)	1.000
Malformation myeloma	5 (0.93)	3 (0.50)	0.4861
Hydrocephalus communicans	4 (0.75)	11 (1.83)	0.1256
Arachnoidal cyst	3 (0.56)	4 (0.66)	1.000
Benign intracranial hypertension	3 (0.56)	2 (0.33)	0.6707
Malformation cranium	2 (0.37)	1 (0.17)	0.604
Arnold chiari malformation	1 (0.19)	1 (0.17)	1.000
Malformation brain	1 (0.19)	1 (0.17)	1.000
**Functional**			
Chronic pain	17 (3.18)	21 (3.49)	0.869
Parkinson disease	14 (2.62)	13 (2.16)	0.6978
Epilepsia	6 (1.12)	4 (0.66)	0.530
Spasticity	3 (0.56)	10 (1.66)	0.0977
Tremor	2 (0.37)	1 (0.17)	0.6039
Myopathia, neuropathia	2 (0.37)	7 (1.16)	0.1848
Chorea Huntington	0	1 (0.17)	1.000
Dystonia	0	1 (0.17)	1.000
**Trauma**			
Traumatic SDH	21 (3.93)	21 (3.49)	0.7538
Traumatic SAH	7 (1.31)	8 (1.33)	1.000
Epidural hemorrhage	5 (0.93)	3 (0.50)	0.486
TBI	3 (0.56)	4 (0.66)	1.000
Skull fracture	2 (0.37)	1 (0.17)	0.6039
Brain contusion	1 (0.19)	3 (0.50)	0.6269
Fracture vertebra	1 (0.19)	0	0.4705
**Peripheral nerve surgery**			
Nerve lesion	1 (0.19)	10 (1.66)	0.0128
Peripheral neurinoma	2 (0.37)	0	0.2212
**Other**			
Abscess	3 (0.56)	5 (0.83)	0.729
Ataxia	2 (0.37)	0	0.221
Spinal hemorrhage	1 (0.19)	0	0.4705
Trigeminus neuralgia	0	1 (0.17)	1.000

N, Number; CNS, central nervous system; ICH, intracranial hemorrhage; subdural hematoma; SAH, subarachnoidal hemorrhage; NPH, normal pressure hydrocephalus; TBI, traumatic brain injury.

**Table 3:** Unplanned readmission in pandemic and pre-pandemic patient groups.

N = 62	Pandemic		Pre-Pandemic		
	Median, Range	N (%)	Median, Range	N (%)	*p*-Value
**Unplanned readmission**		27 (5.05)		35 (5.81)	0.603
Age, yrs.	59; 0–82		59; 2–86		0.514
Gender, female		14 (51.85)		18 (51.43)	1.000
**Index diagnosis group**					
Neoplasm		6 (22.22)		10 (28.57)	0.771
Hydrocephalus/malformation		7 (25.93)		6 (17.14)	0.532
Trauma		2 (7.41)		1 (2.86)	0.575
Vascular		2 (7.41)		8 (22.86)	0.164
Degenerative spine		6 (22.22)		2 (5.71)	0.069
Functional		3 (11.11)		4 (11.43)	1.000
Other		1 (3.70)		4 (11.43)	0.376
Complication, index admission		17 (62.96)		19 (54.29)	0.606
PCCL, index admission	3; 0–5		2; 0–5		0.427
LOS readmission, d	7; 1–36		7; 0–64		0.875
Time admission, readmission d	13; 0–28		13; 3–30		0.927
Reoperation		17 (62.96)		19 (54.29)	0.606

N, number; yrs, years; PCCL, Patient clinical complexity level; LOS, length of stay; d, days.

## Supplementary Materials

The following are available online at https://www.mdpi.com/article/10.3390/ijerph182212034/s1, Table S1: Detailed unplanned readmission reasons for the pandemic and pre-pandemic patient group.

## Abbreviations

CNS: central nervous system; COVID-19: Corona-virus-19; CSF: cerebrospinal fluid; EDH: epidural hematoma; ICD-10-GM: Classification of Diseases and Related Health Problems, 10th Revision, German Modification; ICP: intracranial pressure; LOS: length of stay; NPH: normal pressure hydrocephalus; No.: number; PCCL: patient clinical complexity level; SSI: surgical site infection; SDH: subdural hematoma; SAH: subarachnoidal hemorrhage; TBI: traumatic brain injury.
