# Supplementary material for: Neurosurgical Care during the COVID-19 Pandemic in Central Germany: A Retrospective Single Center Study of the Second Wave
Source: Int J Environ Res Public Health. 2021 Nov 16;18(22):12034. doi: 10.3390/ijerph182212034 (PMC8618904; doi:10.3390/ijerph182212034)
Supplement: Supplementary file 1 [file ijerph-18-12034-s001.zip › ijerph-1439945-supplementary.pdf]

Table S1. Detailed unplanned readmission reasons for the pandemic and pre-pandemic patient group.

N = 62	Pandemic N = 27	Pre-Pandemic N = 35	
**Readmission Causes**	**N (%)**	**N (%)**	***p*-Value**
SSI	5 (18.52)	10 (28.57)	0.3903
Nosocomial infection	5 (18.52)	2 (5.71)	0.223
Recurrent hemorrhage	3 (11.11)	2 (5.71)	0.6450
Shunt dysfunction	2 (7.41)	1 (2.86)	0.5752
Convulsion	1 (3.70)	6 (17.14)	0.1257
Hydrocephalus	1 (3.70)	3 (8.57)	0.6256
Recurrent disk herniation	2 (7.41)	0	0.1856
CSF infection	1 (3.70)	1 (2.86)	0.435
Fall	2 (7.41)	1 (2.86)	0.5752
Malfunction device	1 (3.70)	2 (5.71)	1.000
Pain	1 (3.70)	1 (2.86)	1.000
Neurologic impairment	1 (3.70)	0	0.4355
Stroke	1 (3.70)	0	0.4355
Social indication	0	2 (5.71)	0.5003
Other hemorrhage	1 (3.70)	0	0.4355
Thrombosis	0	1 (2.86)	1.000
Multiple organ failure	0	1 (2.86)	1.000
Electrolyte degradation	0	1 (2.86)	1.000
Ileus	0	1 (2.86)	1.000

SSI, surgical site infection; CSF, cerebrospinal fluid.
